# Hydrogen sulphide ameliorating skeletal muscle atrophy in db/db mice via Muscle RING finger 1 S‐sulfhydration

**DOI:** 10.1111/jcmm.15587

**Published:** 2020-07-07

**Authors:** Fangping Lu, Baoling Lu, Linxue Zhang, JingChen Wen, Mengyi Wang, Shiwu Zhang, Qianzhu Li, Feng Shu, Yu Sun, Ning Liu, Shuo Peng, Yajun Zhao, Shiyun Dong, Dechao Zhao, Fanghao Lu, Weihua Zhang

**Affiliations:** ^1^ Department of Pathophysiology Harbin Medical University Harbin China; ^2^ Department of Infectious The Fourth Hospital of Harbin Medical University Harbin China; ^3^ Department of Cardiology First Affiliated Hospital of Harbin Medical University Harbin China

**Keywords:** diabetes mellitus, hydrogen sulphide, Muscle RING finger 1, skeletal muscle atrophy, S‐sulfhydration modification

## Abstract

Muscle atrophy occurs in many pathological states, including cancer, diabetes and sepsis, whose results primarily from accelerated protein degradation and activation of the ubiquitin‐proteasome pathway. Expression of Muscle RING finger 1 (MuRF1), an E3 ubiquitin ligase, was increased to induce the loss of muscle mass in diabetic condition. However, hydrogen sulphide (H_2_S) plays a crucial role in the variety of physiological functions, including antihypertension, antiproliferation and antioxidant. In this study, db/db mice and C2C12 myoblasts treated by high glucose and palmitate and oleate were chose as animal and cellular models. We explored how exogenous H_2_S attenuated the degradation of skeletal muscle via the modification of MuRF1 S‐sulfhydration in db/db mice. Our results show cystathionine‐r‐lyase expression, and H_2_S level in skeletal muscle of db/db mice was reduced. Simultaneously, exogenous H_2_S could alleviate ROS production and reverse expression of ER stress protein markers. Exogenous H_2_S could decrease the ubiquitination level of MYOM1 and MYH4 in db/db mice. In addition, exogenous H_2_S reduced the interaction between MuRF1 with MYOM1 and MYH4 via MuRF1 S‐sulfhydration. Based on these results, we establish that H_2_S prevented the degradation of skeletal muscle via MuRF1 S‐sulfhydration at the site of Cys44 in db/db mice.

## 1 INTRODUCTION

The global prevalence of type 2 diabetes is rapidly growing. Chronic type 2 diabetes causes decreased muscle mass, which leads to disability and morbidity in type 2 diabetes patients.^1^ Rising evidence has demonstrated that type 2 diabetes and muscle atrophy develop synchronously. Especially, the older type 2 diabetic patients cause muscle atrophy faster than those without diabetic patients, which are positively correlated with death rates in these populations.^2^, ^3^ Type 2 diabetes is involved in metabolic dysfunction, such as hyperglycaemia, hyperlipidaemia and hypoinsulinaemia, which may be contribute to loss of muscle. However, the exact molecular mechanism of muscle atrophy in type 2 diabetes is still unclear.

Reactive oxygen species (ROS) plays a crucial role in the pathogenesis of metabolic diseases. In diabetic state, hyperglycaemia and hyperlipidaemia increase oxidative stress, including endoplasmic reticulum stress (ER stress). ER is responsible for proteins mature from folding and secretion. When overexcessive misfold proteins are generated, ER initiates unfold protein response (UPR). If a certain threshold is exceeded, it can cause the endoplasmic reticulum‐associated degradation (ERAD).^4^, ^5^ Protein degradation by the ubiquitin‐proteasome system may destroy the structure of the tissues.

Hydrogen sulphide (H_2_S), as a new endogenous gas after nitric monoxide (NO) and carbon monoxide (CO), plays an important role in antihypertension, antioxidant and anti‐inflammation. In mammalian cells, the biosynthesis of H_2_S is generated by pyridoxal‐5‐phyosphate‐depordent enzymes, including cystathionine‐γ‐lyase (CSE), cystathionine‐β‐synthetase (CBS) and 3‐mercaptopyruvate sulphurtransferase (3‐MST).^6^, ^7^ Our previous study has revealed H_2_S could promote clearance of ubiquitin aggregates, which contributes to its antioxidative effects in DCM.^8^ Since the pathogenesis of skeletal muscle atrophy in type 2 diabetes is complex, the exact mechanism by which H_2_S may protect against the loss of muscle mass is still not thoroughly explained. The aim of the present study was to explore whether H_2_S could attenuate skeletal muscle atrophy to regulate MuRF1.

## 2 MATERIALS AND METHODS

### 2.1 Animal model and treatment

The animal experiment was divided into three groups: control, db/db and db/db + NaHS. All animals (db/db mice, n = 90) were purchased from the Animal Laboratory Centre of Nanjing University and their age‐matched non‐diabetic C57BL/6 mice as a control. All animals were kept in a constant temperature, and environment, water and food were freely consumed. Half of the db/db mice were injected with NaHS (80 μmol/kg) as a treatment group every day for 6, 12 and 20 week.^9^, ^10^ At the end of the treatment, the mice, fasted for 12 hours, were anesthetized via injection with 4% chloral hydrate. Gastrocnemius muscle was removed from mice, frozen in liquid nitrogen and stored at −80℃ for the protein expression experiment. Small pieces of the skeletal muscle tissues were fixed with 4% paraformaldehyde for biochemical experiments. All animal experiments were performed by the Guide for the Care and Use of Laboratory Animals published by the China National Institutes of Health and approved by the Animal Care Committees of Harbin Medical University, China.

### 2.2 Cell culture and treatment

C2C12 myoblasts were cultured in DMEM supplemented with 10% foetal bovine serum (FBS) with penicillin and streptomycin at 37ºC in 5%CO_2_ and differentiated in DMEM supplemented with 2% horse serum. The differentiated C2C12 myoblasts were randomly divided into the following groups: control (high glucose, 25 mmol/L), HG + palmitate + oleate (high glucose, 40 mmol/L, palmitate, 200 μmol/L, oleate, 200 μmol/L), HG + palmitate + oleate + NaHS (NaHS, 100 μmol/mL), HG + palmitate + oleate + PPG (DL‐propargylglycine, a CSE inhibitor, 10 nmol/L), HG + palmitate + oleate + dithiothreitol (DTT, an inhibitor of disulphide bond, 1 mmol/L), HG + palmitate + oleate + N‐acetylcysteine (NAC, an inhibitor of ROS, 100 μmol/L), HG + palmitate + oleate + 4‐phenylbutyrate (4‐PBA, an inhibitor of endoplasmic reticulum stress, 5 mmol/L), HG + palmitate + oleate + thapsigargin (tg, an inducer of endoplasmic reticulum stress, 100 μmol/L), HG + palmitate + oleate + MG132 (an inhibitor of 26S proteasome, 20 μmol/L) and HG + palmitate+oleate + PYR41 (an inhibitor of ubiquitin‐activating enzyme (E1), 3 μmol/L). All the groups were treated for 48 hours.

### 2.3 Western blot analysis

The proteins of the skeletal muscle tissue and C2C12 myoblasts were extracted with RIPA and PMSF (100:1). After centrifugation at 4°C, the supernatant was collected for protein sample concentration determination using the BCA Protein Assay kit (Beyotime, Shanghai, China). Protein lysates from each sample of cells and tissues pass separation by electrophoresis on SDS‐PAGE and electrotransfer to PVDF Membrane (Millipore, Bedford, MA, USA). After blocking for 1.5 hour, the membranes were incubated with primary antibodies against anti‐GAPDH (1:1000; ProteinTech Group, Wuhan, China), anti‐CAT (1:1000; ProteinTech Group), anti‐SOD (1:1000; ProteinTech Group), anti‐Bip (1:1000; ProteinTech Group), anti‐p‐PERK (Thr^981^, 1:500; Santa Cruz Biotechnology, Santa Cruz, CA, USA), anti‐PERK (1:1000; ProteinTech Group), anti‐ATF4 (1:1000; ProteinTech Group), anti‐p‐eIF2α (Ser^51^, 1:1000; ProteinTech Group), anti‐eIF2α (1:1000; ProteinTech Group), anti‐ubiquitin (1:1000; ProteinTech Group), anti‐MuRF1 (1:1000; ProteinTech Group), anti‐MYOM1 (1:1000; ProteinTech Group), anti‐MYH4 (1:1000; ProteinTech Group) and anti‐CSE (1:1000; ProteinTech Group) at 4℃ overnight and then incubated with secondary antibody diluted at 1:5000 conjugated to horseradish peroxidase (ProteinTech TM, Wuhan, Hubei, China) at room temperature for 1 hour. The results were detected by enhanced chemiluminescence reagent (Santa Cruz Biotechnology) and then analysed by AlphaView.SA.

### 2.4 Immunoprecipitation

Dilute each sample to 2 μg/μL and anti‐MuRF1 antibodies (following to manufacturer's instructions) and then incubate with IgG‐conjugated agarose beads (50 μL), together with rotation at 4°C overnight. Centrifuge and wash three times with RIPA lysis buffer containing 1% PMSF to collect precipitate, analysis of protein interactions with Western blot.

### 2.5 Immunofluorescence

Fresh tissues were removed and were positioned vertically on the cryostat. Sections were mounted on slides and fixed with 4% paraformaldehyde for 30 minutes, washed with phosphate buffered saline (PBS) for 10 minutes and then permeabilized in PBS/Triton (0.2%) for 10 minutes. Washed three more times with PBS, avoided staining with DHE or 7‐azido‐4‐methylcoumarin (1:1000) probe, DHE (1:1000) probe, phalloidin (1:1000) probe incubated at 37°C for 30 minutes, washed three times with PBS for 15 minutes and incubated with 1:1000 dilution of DAPI solution at 37°C for 10 minutes. Then, washed for 15 minutes. Images were captured using confocal microscopy (40× magnification).

### 2.6 Muscle fibre atrophy

The gastrocnemius muscle was removed from the hindlimbs of mice. One part was used to directly observe the size of gastrocnemius muscle, the other part was used to detect the degree of muscle atrophy by LAMININ staining, and the measure of muscle fibre atrophy was performed as reported previously.^11^ Frozen section with phalloidin (1:1000) probe staining also detected the muscle fibre atrophy, according to the instruction.

### 2.7 ROS level analysis

C2C12 myoblasts were extracted, and the expression of CAT and SOD protein in tissues and cells was detected by Western blot; the other part was inoculated in the 24‐well plate to detect the ROS level by DHE staining. According to the instruction, we also used SOD and CAT diagnostic reagent kits (Nanjing Jiancheng Bioengineering) to detect the activity of SOD and CAT.

### 2.8 Biotin switch to detect S‐sulfhydration modification

Biotin switch as described in previous study.^12^ Briefly, the differentiated C2C12 myoblasts were homogenized in HEN buffer containing 250 mmol/L Hepes‐NaOH (pH 7.7), 1 mmol/L EDTA and 0.1 mmol/L neocuproine, supplemented with 100 mmol/L deferoxamine and centrifuged at 13 000 *g* for 30 minutes at 4°C. C2C12 myoblasts lysates were added to four times blocking buffer (HEN buffer with 2.5% SDS and 20 mmol/L methyl methanethiosulfonate) at 50°C for 20 minutes with frequent vortexing. Methyl methanethiosulfonate was later removed by four times acetone, and the proteins were precipitated at −20°C for 60 minutes. After acetone removal, proteins were resuspended in HENS buffer with 1% SDS. To the suspension was added 4 mmol/L biotin‐HPDP in dimethyl sulphoxide. After incubation for 3 hours at 25°C, biotinylated proteins were precipitated by streptavidin‐agarose beads, which were later washed with HENS buffer. The biotinylated proteins were eluted by SDS‐polyacrylamide gel electrophoresis (SDS‐PAGE) sample buffer and subjected to Western blot analysis using antibodies against MuRF1.

### 2.9 Detection of intracellular polysulphide level

The differentiated C2C12 myoblasts were inoculated in the 24‐well plate to detect the intracellular polysulphide level by fluorescent probe, SSP4, with slight modifications.^13^ Briefly, cells were loaded with SSP4 (50 μmol/L) in a serum‐free DMEM medium containing 0.003% Cremophor EL for 15 minutes at 37°C in the dark. After being washed, SSP4 was detected using the fluorescence microscope (Olympus, Shenzhen, China, XSZ‐D2).

### 2.10 Computational method

To date, the three‐dimensional (3D) structure of MuRF1 has not been resolved; therefore, homology modelling, a promising tool to predict protein structure based on a template containing similar sequence, was applied in this study using Phrye2. The sequence of MuRF1 was obtained from Uniprot (RRID:SCR_002380). According to a sequence alignment by BLAST (RRID:SCR_004870), the crystal structure and active site of MuRF1 were calculated by the website of protein plus (RRID:SCR_005375).

### 2.11 Point mutation of MuRF1

Adenoviruses expressing GFP and MuRF1‐GFP were purchased from Cyagen Biosciences Inc (Guangzhou, China). The full‐length MuRF1 of mouse was mutated by a single cysteine site. This article is protected by copyright. All rights reserved 44 to alanine and GFP cDNA was inserted into pM vector (Cyagen Biosciences, Guangzhou, China) between the Kozak and T2A sites. The adenovirus was added to the differentiated C2C12 myoblasts, transfected for 4‐6 hours and then was added new fresh medium. After 24 hours of transfection, the dosing medium was added according to different conditions, and then, the proteins were collected and analysed by Western blotting.

### 2.12 Statistical analyses

Data were presented as mean ± SD. Data were compared using a one‐way ANOVA. Results were analysed by using Prism 5 (GraphPad Software, San Diego, CA, USA). Tukey's post hoc test was used for determining the significance, and the threshold of *P* less than 0.05 was designated as statistically significant.

## 3 RESULTS

### 3.1 H_2_S level and CSE expression in skeletal muscle in db/db mice

**Figure 1:**
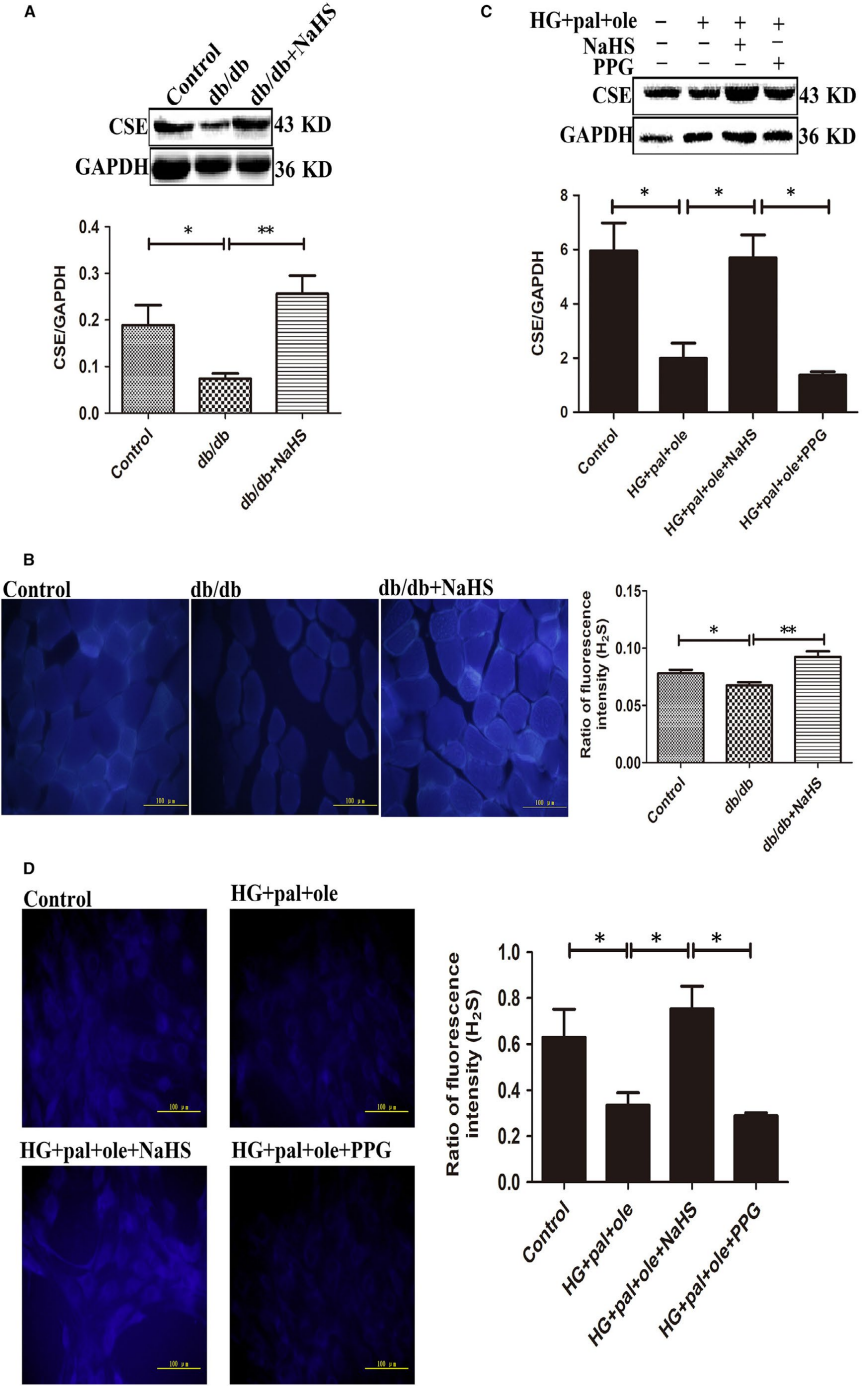
CSE expression and H_2_S level was detected in skeletal muscle in db/db mice and C2C12 myoblasts. A, C, CSE expression was examined by Western blotting. B, D, 7‐azido‐4‐methylcoumarin staining was used to measure intracellular H_2_S level. Data shown are means ± SD, n = 4, *P < 0.05, **P < 0.01. Significantly different as indicated. One‐way ANOVA. CSE, cystathionine‐r‐lyase; H_2_S, hydrogen sulphide

In this study, we first detected the blood glucose and TAG content in plasma, and we found that the blood glucose and TAG content in db/db mice significantly increased compared to control and treated by NaHS groups (Figure S1). Our previous study demonstrated that level of H_2_S and expression of CSE reduced in cardiac tissues in db/db mice.^8^, ^14^ Our results showed that expression of CSE was declined at 20 weeks in skeletal muscle of db/db mice, compared to control group; however, there was no significant change at 6 and 12 weeks (Figure S2). Administration of NaHS could restore expression of CSE at 20 weeks (Figure 1A). In the following experiments, we chose the gastrocnemius at 20 weeks. Next, we also examined the level of H_2_S by 7‐azido‐methylcoumarin staining, a specific fluorescent probe for H_2_S. We found that the level of H_2_S in db/db mice was significantly lower than that in control and NaHS groups (Figure 1B). We also tested the level of H_2_S and CSE expression in C2C12 myoblasts under high glucose and palmitate and oleate condition to mimic type 2 diabetic model. Our results showed that the level of H_2_S and expression of CSE was significantly decreased (Figure 1C,D) in HG + pal + ole and PPG (an inhibitor of CSE) groups. These results were in accordance with the data in db/db mice.

### 3.2 Hydrogen sulphide attenuated skeletal muscle mass atrophy in db/db mice

**Figure 2:**
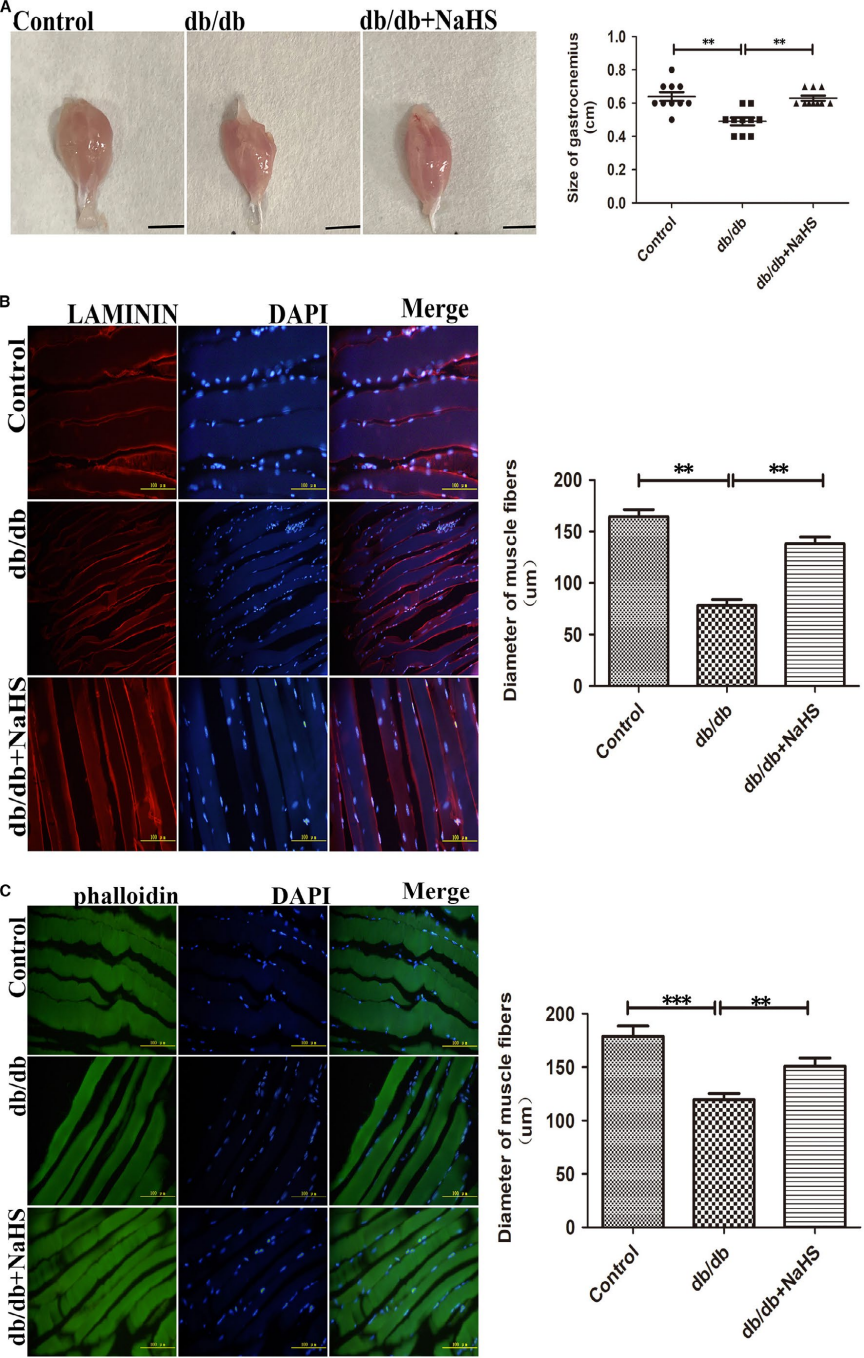
Exogenous H_2_S reduced the loss of skeletal muscle mass in db/db mice. A, Representative image of skeletal muscle isolated from control, db/db mice and db/db mice with the treatment of NaHS. B, C, Skeletal muscle structure was detected by phalloidin (green) staining of F‐actin and LAMININ (red) staining. Data shown are mean S ± SD, n = 10, **P < 0.01, ***P < 0.001. Significantly different as indicated. One‐way ANOVA. H_2_S, hydrogen sulphide

To determine whether the reduction of H_2_S correlates with the loss of skeletal muscle mass, we detected the size of different muscle groups. Our results showed that the gastrocnemius in db/db mice exhibited ~25% reduction in muscle size, compared to control and exogenous H_2_S group (Figure 2A). We also found that this reduction in muscle size in db/db mice was accompanied by a significant reduction in diameter of muscle fibre by phalloidin and LAMININ staining, compared to control and db/db mice treated by NaHS (Figure 2B,C).

### 3.3 Exogenous H_2_S reduced ROS production and ameliorated ER stress in vivo and in vitro

**Figure 3:**
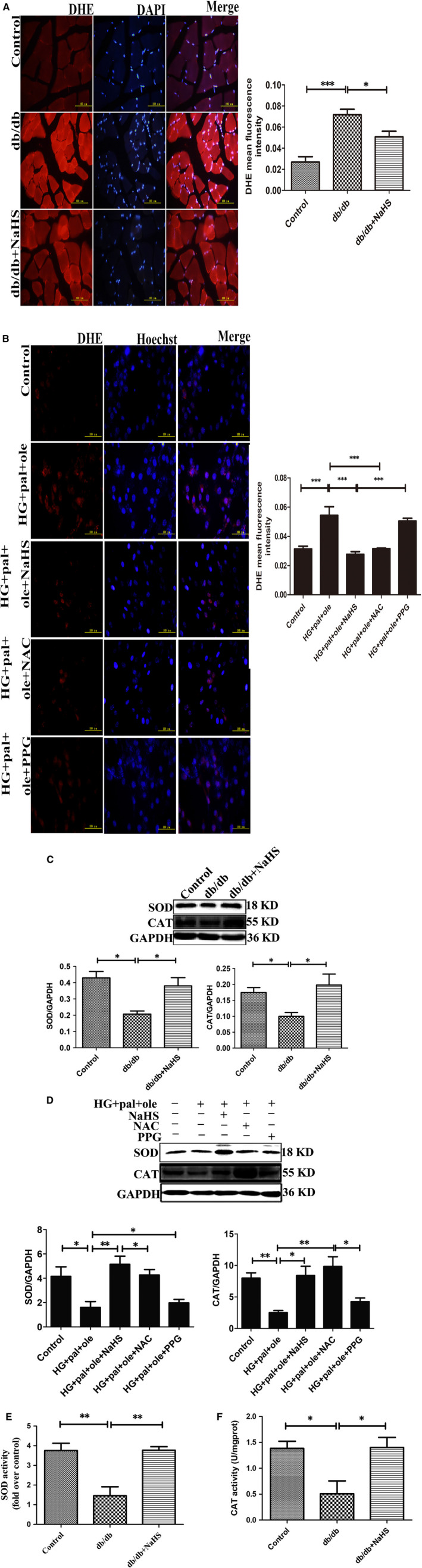
Exogenous H_2_S decreased oxidative stress induced diabetic atrophied muscle. A, B, Superoxide generation was measured in frozen muscle sections and C2C12 myoblasts of control and db/db and db/db mice with the treatment of NaHS and C2C12 myoblasts treated by NAC and PPG using dihydroethidium staining. C, D, Expression of SOD and CAT in animal and cellular model were tested by Western blot. E, F, The activities of SOD and CAT were measured using spectrophotometric‐based assay. G, H, Western blotting analyses the expression of protein markers of ER stress in animal and cellular model. Values are mean ± SD. n = 5, *P < 0.05, **P < 0.01, ***P < 0.001. Significantly different from corresponding control values. H_2_S, hydrogen sulphide

Increasing evidence has confirmed that H_2_S has antioxidant effects. We assessed ROS production in skeletal muscle and C2C12 myoblasts using DHE staining. Production of ROS was increased in db/db and HG + pal + ole groups compared to control, NaHS and NAC (an ROS scavenger) groups, whereas PPG could increase ROS production (Figure 3A,B). We also examined the expression and activity of SOD and CAT. Our results showed that expression of SOD and CAT was obviously decreased in db/db and HG+pal+ole groups, compare to control, NaHS and NAC groups (Figure 3C,D). In line with this, activity of SOD and CAT in db/db group was significantly decreased, compared to control and db/db mice with the treatment of NaHS (Figure 3E,F).

To explore whether H_2_S could attenuate ER stress in db/db mice, we analysed the expression of ER stress‐associated proteins including Bip, ATF4, p‐PERK (Thr^981^)/PERK and p‐eIF2α (Ser^51^)/eIF2α. These proteins were up‐regulated in db/db mice, but exogenous H_2_S could reduce these protein levels (Figure 3G). To further investigate H_2_S could inhibit ER stress in vitro, above proteins were also assayed in C2C12 myoblasts. Our data showed that exogenous H_2_S and 4PBA (an inhibitor of ER stress) could restore the expression of ER stress‐associated proteins. Tg (an activator of ER stress) and HG and palmitate and oleate could elevated these protein levels (Figure 3H). These results suggested that exogenous H_2_S could suppress ROS production and the occurrence of ER stress.

### 3.4 H_2_S reduced the degradation of MYOM1 and MYH4 in db/db mice

**Figure 4:**
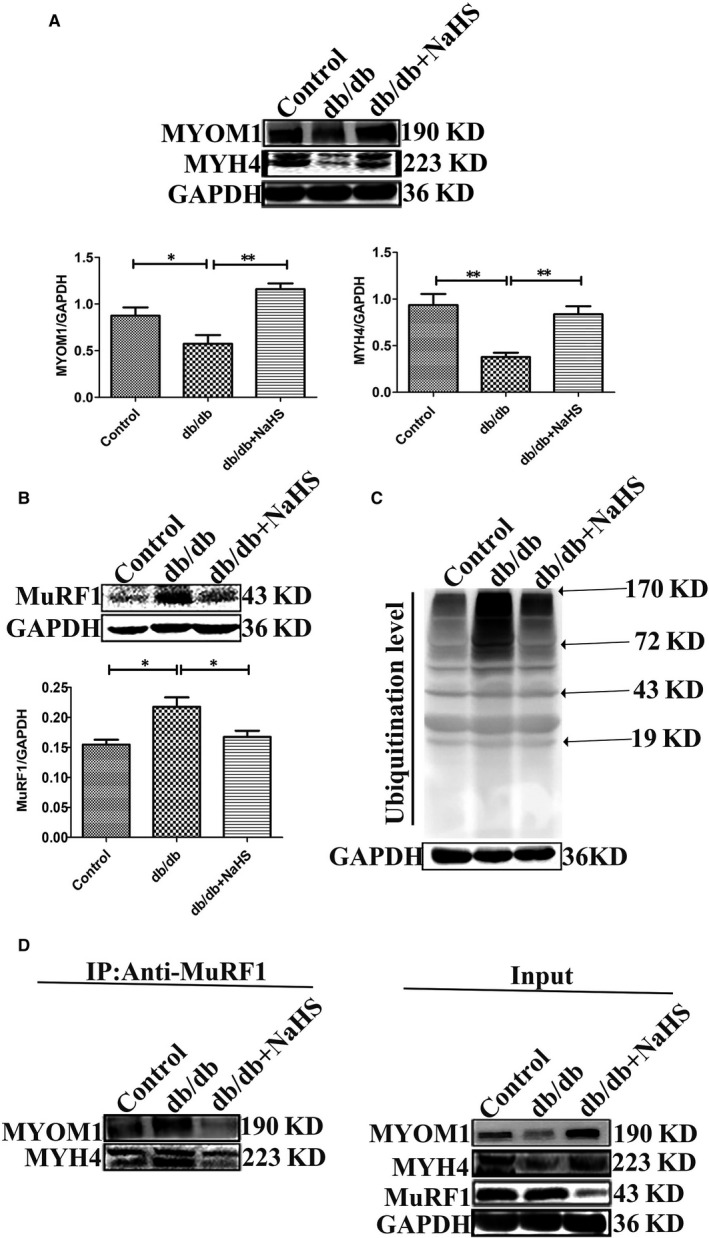
Exogenous H_2_S inhibited the skeletal muscle degradation. A, Expression of skeletal muscle structural proteins of MYOM1 and MYH4 were examined by Western blotting. B, MuRF1 expression was tested by Western blotting. C, The ubiquitination of skeletal muscle was measured by Western blot. D, Skeletal muscle lysates was immunoprecipitated with MuRF1 antibody and then immunoblotted with antibodies for MYOM1 and MYH4. Data shown are means ± SD, n = 5, *P < 0.05, **P < 0.01. Significantly different as indicated. One‐way ANOVA. H_2_S, hydrogen sulphide; MuRF1, Muscle RING finger 1

Figure 2A,B illustrated that skeletal muscle in db/db mice was atrophic. Further analysis showed that the protein level of MYOM1 and MYH4, the structural protein of skeletal muscle, was significantly decreased in db/db group, compared to control and db/db mice treated by NaHS (Figure 4A). Some studies have revealed that ER stress can initiate ubiquitin‐proteasome system.^15^, ^16^ Thus, we analysed the E3 ubiquitin ligase of MuRF1 is related to skeletal muscle degradation.^17^, ^18^ Our results revealed that expression of MuRF1 in db/db group was significantly increased, compared to control and with the treatment of NaHS groups (Figure 4B). We also detected the ubiquitylation level in skeletal muscle in different groups. Our results showed that the ubiquitylation level in db/db group was obviously higher than that in control and db/db mice treated by NaHS groups (Figure 4C). Furthermore, our immunoprecipitation results showed that exogenous H_2_S reduced the interaction between MuRF1 with MYOM1 and MYH4 (Figure 4D). These results demonstrated that H_2_S could decrease skeletal muscle degradation through regulating MuRF1.

### 3.5 H_2_S attenuated MYOM1 and MYH4 degradation through reducing MuRF1 expression in vitro

**Figure 5:**
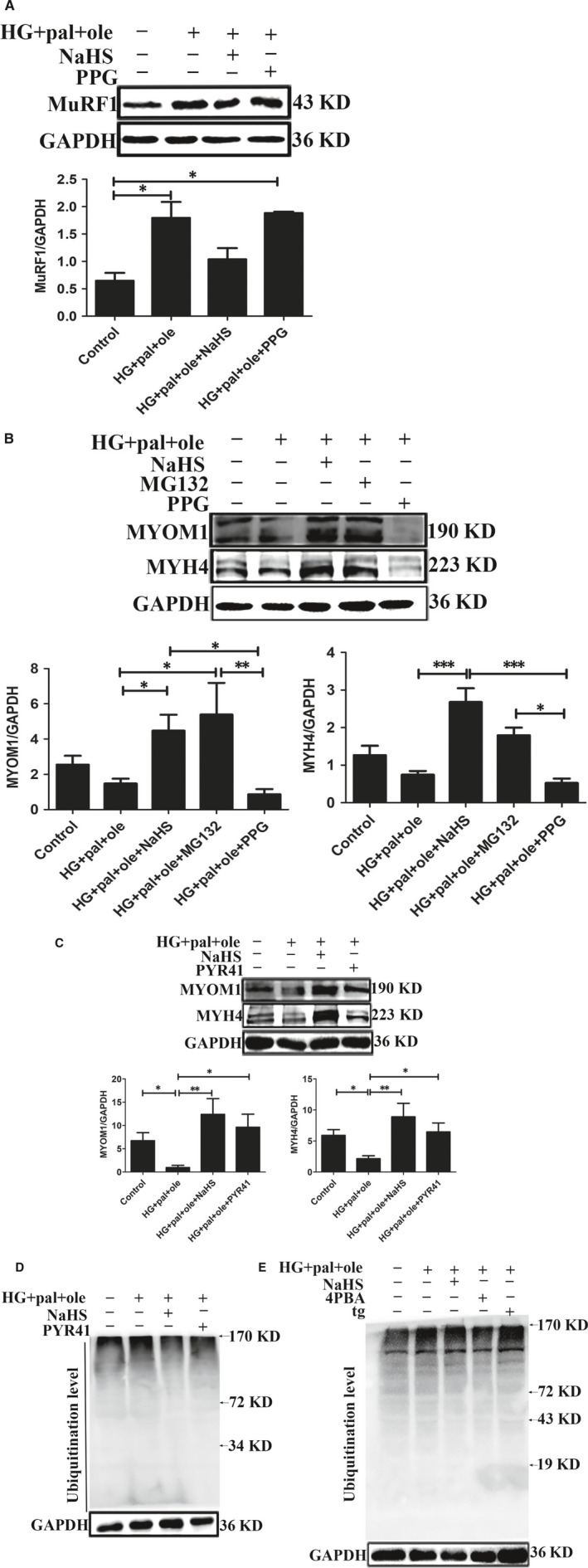
Effect of exogenous H_2_S on ubiquitination level of skeletal muscle structural proteins in C2C12 myoblasts. A, Expression of MuRF1 exposed to high glucose and palmitate and oleate condition for 48 hours. B, C, C2C12 myoblasts treated by MG132 (20 μmol/L, an inhibitor of proteasome) for 30 minutes or PYR41 (3 μmol/L, an inhibitor of ubiquitin‐activating enzyme) for 1 hour. Expression of MYOM1 and MYH4 was measured by Western blotting. D, E, The ubiquitination level was detected by Western blotting, with the administration of 4‐PBA (5 mmol/L, an inhibitor of ER stress), tg (100 μmol/L, an activator of ER stress) and PYR41. Data shown are means ± SD, n = 3, *P < 0.05, **P < 0.01. Significantly different as indicated. One‐way ANOVA. H_2_S, hydrogen sulphide; MuRF1, Muscle RING finger 1

To further analyse whether H_2_S was involved in decreasing MYOM1 and MYH4 degradation through regulating MuRF1 expression, we first examined expression of MuRF1 in C2C12 myoblasts. Our results showed that expressions of MuRF1 in HG + pal + ole and PPG groups were significantly increased, compared to control and treated by NaHS groups (Figure 5A). In our experiments, two inhibitors of ubiquitin‐associated enzymes, MG132 (26S proteasome inhibitor) and PYR41 (E1 ubiquitin‐activating enzyme inhibitor), were used to treat C2C12 myoblasts under high glucose and palmitate and oleate condition. We found that expressions of MYOM1 and MYH4 with the treatment of NaHS, MG132 and PYR41 were significantly increased, compared to HG + pal + ole and PPG groups (Figure 5B,C). Moreover, the ubiquitination level was declined, when PYR41 and NaHS were used in C2C12 (Figure 5D). In addition, the ubiquitination level was increased by using tg, whereas 4‐PBA and NaHS could decrease the ubiquitination level in C2C12 (Figure 5E). These results demonstrated that exogenous H_2_S could decrease the ubiquitination level in vitro.

### 3.6 H_2_S regulated MuRF1 by S‐sulfhydration in myoblasts cells under high glucose and palmitate and oleate condition

**Figure 6:**
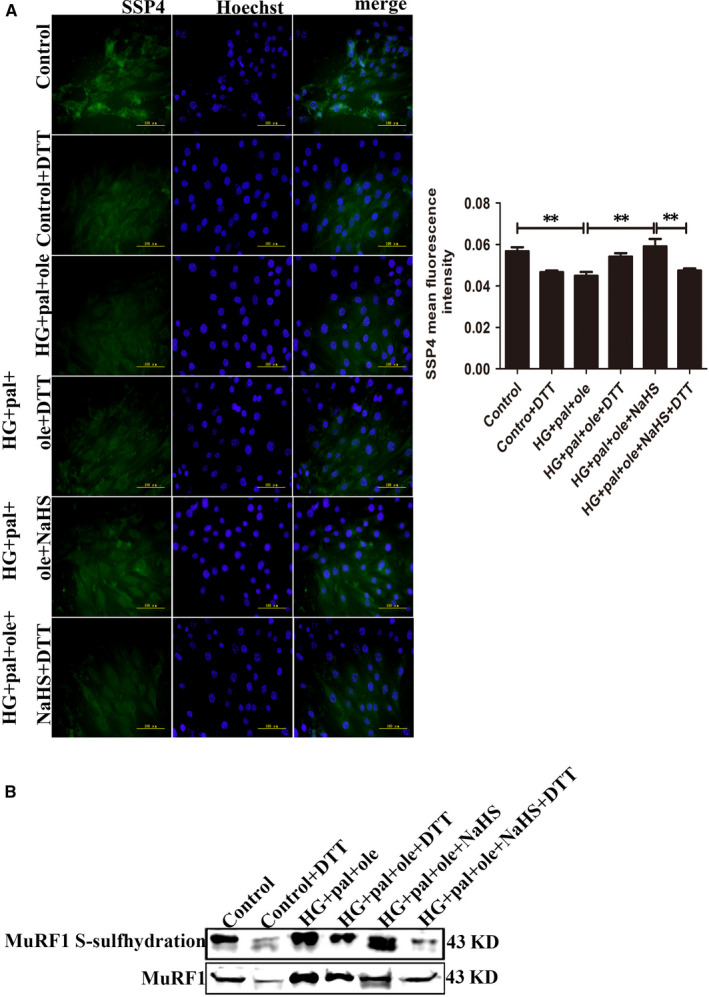
Detection of intracellular polysulphide level in C2C12 myoblasts. A, Fluorescent probe, SSP4 detected polysulphide level in C2C12 myoblasts, DTT (1 mmol/L, reducing agent). B, S‐sulfhydration on MuRF1 was detected with biotin switch assay. Data shown are means ± SD, n = 3, **P < 0.01. Significantly different as indicated. One‐way ANOVA. DTT, dithiothreitol; MuRF1, Muscle RING finger 1

Recent evidence has demonstrated that H_2_S could modify target proteins by S‐sulfhydration, a new way of post‐translation modification, which effects on the activities of target proteins.^19^ We detected the intracellular production of polysulphide using SSP4, a fluorescent probe. Dithiothreitol (DTT), a reducing agent to reverse sulfhydration, was used. The results showed that NaHS could increase SSP4 fluorescent intensity, suggesting polysulphide could be increased after H_2_S application (Figure 6A). To further explore whether H_2_S modified MuRF1 by S‐sulfhydration, we analysed the S‐sulfhydration protein of MuRF1 using biotin switch assay, and we found that H_2_S could regulate MuRF1 by S‐sulfhydration. DTT could decrease the modification of MuRF1 S‐sulfhydration (Figure 6B).

### 3.7 H_2_S modified MuRF1 Cys44 site to attenuate the structure protein degradation

**Figure 7:**
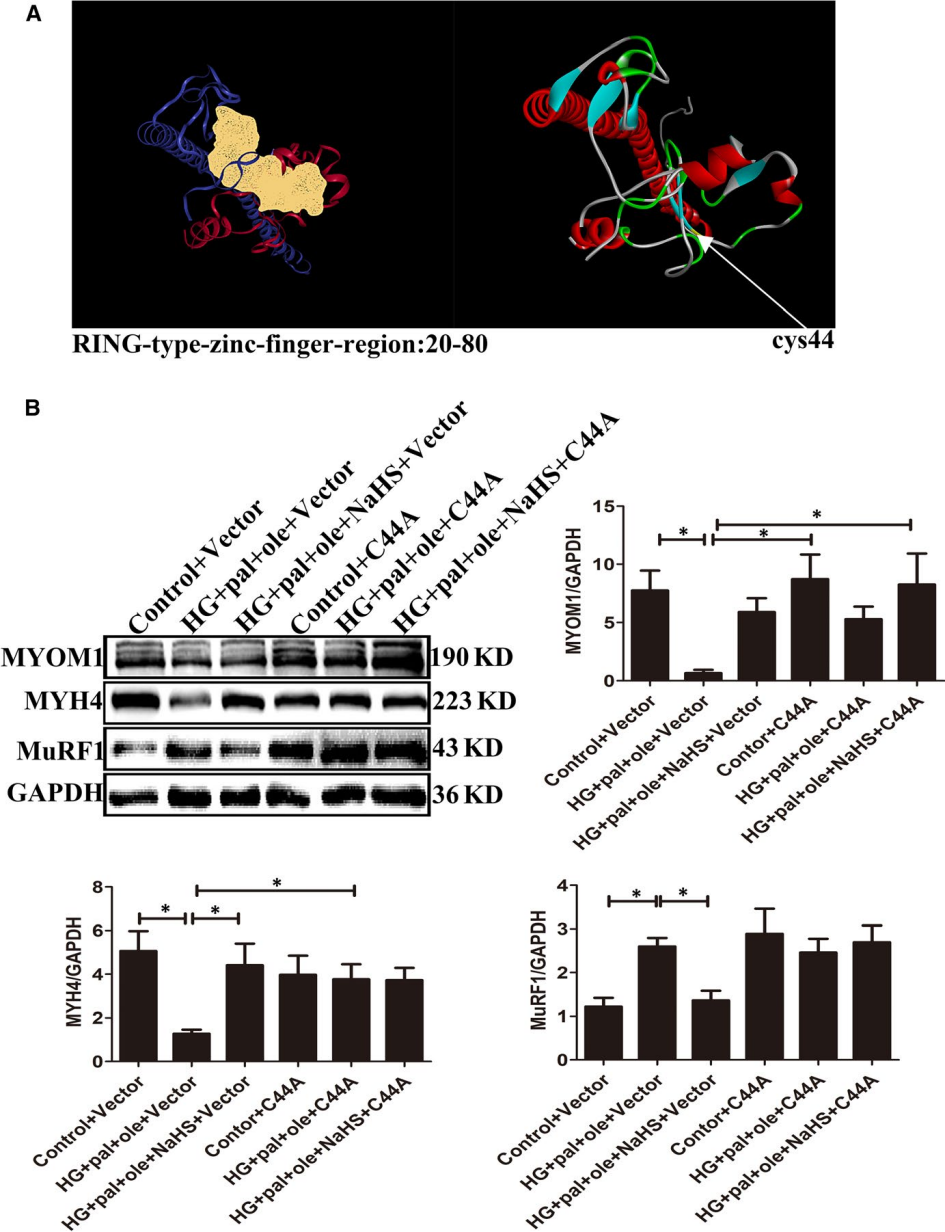
Mutation of MuRF1 Cys44 could attenuate skeletal muscle degradation under high glucose and palmitate and oleate condition. A, The active centre of MuRF1 was predicted by computational method. B, After transfection of MuRF1 Cys44 and control vector for 24 h, expression of MYOM1, MYH4 and MuRF1 was analysed by Western blot. Data shown are means ± SD, n = 4, *P < 0.05. Significantly different as indicated. One‐way ANOVA. MuRF1, Muscle RING finger 1

Muscle RING finger 1 is composed of 350 amino acids, including 17 cysteine residues. MuRF1 contains a RING‐type zinc finger domain, which is associated with ubiquitination modification. We used bioinformatic methods to predict its structure. We found that 23‐79 amino acid residues were its RING‐type zinc finger domain (Figure 7A). Cys44 is located in the active centre. Based on our analysis, we mutated Cys44 in RING‐type zinc finger domain to alanine. Mutant of MuRF1 Cys44 was transfected into C2C12 myoblasts. The expressions of MYOM1 and MYH4 were increased after transfection with the mutation of MuRF1 Cys44, compared to vector group with the treat of high glucose and palmitate and oleate group (Figure 7B). These results suggested that H_2_S could increase MuRF1 S‐sulfhydration and decrease MYOM1 and MYH4 degradation.

## 4 DISCUSSION

Although diabetes affects almost all tissues of the body, including diabetic cardiovascular diseases, renal failure and blindness, diabetes decreasing muscle mass and strength result in increasing risk of disability, which has been paid enough attention.^20^ Aging and Body Composition (Health ABC) study revealed that patients with uncontrolled diabetes, age 70‐80 years, showed about 25% mortality rate if they were obvious skeletal muscle atrophy.^3^, ^21^ Thus, it is essential to identify the intracellular mechanisms to explain skeletal muscle atrophy. In this current study, we demonstrated that (a) H_2_S generation was decreased in db/db mice; (b) exogenous H_2_S reduced the ubiquitination level of MYOM1 and MYH4 in skeletal muscle of db/db mice; and (c) MuRF1 was modified by S‐sulfhydration with administration of exogenous H_2_S to reduce interaction between MuRF1 with MYOM1 and MYH4.

Mounting evidence has illustrated that oxidative stress plays a crucial role in the onset of diabetes and the development of its complications in various animal models.^22^ Overexcessive ROS accumulation may enhance the aggregation of misfolding proteins in the ER, which is called as ER stress. Therefore, ER stress is inferred by activation of the unfolded protein response (UPR). The UPR sensor PERK (PKR‐like eukaryotic initiating factor α kinase), ATF4 (activating transcription factor‐4) and IRE1 (inositol requiring enzyme 1) are involved in UPR. Under physiological states, these three proteins are capped by chaperone Bip and inhibit its activities.^23^ PERK is an ER resident transmembrane serine/threonine kinase, which autophosphorylates to release Bip and phosphorylates the translation initiation factor eIF2α, and eIF2α could regulate the expression of redox control genes.^24^ ATF4 (another ER stress senor) can directly react to abnormally oxidative condition in the ER, because of reduction of disulphides on the luminal surface of ATF4.^25^, ^26^ Our present study demonstrated that hyperglycaemia and hyperlipidaemia enhanced ROS production and elevated ER stress in skeletal muscle of db/db mice. The biomarkers of ER stress, including Bip, p‐PERK, p‐eIF2α and ATF4, were obviously increased in db/db mice and C2C12 myoblasts exposed to high glucose, palmitate and oleate.

Recent evidence suggests that the accumulation of unfolded or misfolded proteins in the ER causes ER stress, which leads to the cytosolic proteasomal degradation of misfolded proteins. ER stress activating ubiquitination proteasome system (UPS) seems to play the predominant degradation.^27^, ^28^ UPS includes four components ubiquitin, the ubiquitination machinery, proteasome and the deubiquitinase (DUBs).^29^ UPS is involved in the various biological processes, for example, the regulation of protein misfolding and endoplasmic reticulum‐associated degradation.^30^ A number of studies have been suggested that in the process of diabetes, the alterations in proteasome activity have been associated with tissue injuries, including diabetic cardiomyopathy, diabetic blindness and atherosclerosis.^31^ Skeletal muscle fibres maintain a continual state of protein replaced through dynamic balance between protein synthesis and degradation.^32^, ^33^ MuRF1, a muscle specific E3 ligase ubiquitin ligase, is an atrophy‐associated gene, which is transcriptionally following denervation; thus, this process associated with diabetes.^34^, ^35^, ^36^ MuRF1 is located in the M‐line region and colocalizes with action one of major components of the Z‐disc in the cardiac and skeletal muscle.^37^ In our study, we found that hyperglycaemia and hyperlipidaemia up‐regulate the expression of MuRF1 in skeletal muscle in db/db mice and C2C12 myoblasts, which plays a crucial role in loss of muscle mass. The ubiquitination level of two skeletal muscle structural proteins, MYOM1 and MYH4, was obviously elevated in db/db mice and C2C12 myoblasts. Administration of MG132, a proteasome inhibitor, decreased the degradation of MYOM1 and MYH4 in C2C12 myoblasts under HG + pal + ole conditions. Taken together, our results confirmed that hyperglycaemia and hyperlipidaemia could induce the activation of UPS to be involved in skeletal muscle atrophy.

Hydrogen sulphide, as a new gas transmitter, is involved in antihypertension, antiproliferation and antioxidation. In this study, we also demonstrated that exogenous H_2_S could reduce ROS production, which was induced by hyperglycaemia and hyperlipidaemia. Mounting evidence has reported that H_2_S takes part in reducing cysteine disulphide bond, while polysulphides are involved in the addition of bound sulphate sulphur (sulfhydrate) to cysteine residues to modify the target proteins. S‐sulfhydration is a post‐translational modification on specific cysteine residues of target proteins by H_2_S.^38^, ^39^ In our study, we found that NaHS increased the S‐sulfhydration of MuRF1. In addition, exogenous H_2_S inhibited the interaction between MuRF1 with MYOM1 and MYH4. When MuRF1 was mutated at Cys44, the S‐sulfhydration of MuRF1 was decreased and inhibited the interaction between MuRF1 with MYOM1 and MYH4.

In summary, our findings have demonstrated that H_2_S modified MuRF1 S‐sulfhydration at Cys44 to inhibit MYOM1 and MYH4 ubiquitination level to reduce the loss of muscle mass in skeletal muscle in db/db mice. Our study provided a new treatment strategy for diabetic skeletal muscle atrophy.

## CONFLICT OF INTERESTS

The authors declare that they have no conflict of interests.

## AUTHOR CONTRIBUTIONS

**Fangping Lu:** Conceptualization (equal); data curation (equal); methodology (equal); project administration (equal); writing‐original draft (equal). **Baolin Lu:** Conceptualization (equal); data curation (equal); methodology (equal); project administration (equal); writing‐original draft (equal). **Linxue Zhang:** Investigation (equal); visualization (equal). **Jingchen Wen:** Formal analysis (equal); software (equal). **Mengyi Wang:** Formal analysis (equal); software (equal). **Shiwu Zhang:** Formal analysis (equal); software (equal). **Qianzhu Li:** Formal analysis (equal); software (equal). **Feng Shu:** Formal analysis (equal); software (equal). **Yu Sun:** Formal analysis (equal); software (equal). **Ning Liu:** Investigation (equal). **Shuo Peng:** Investigation (equal). **Yajun Zhao:** Supervision (equal). **Shiyun Dong:** Supervision (equal). **Dechao Zhao:** Supervision (equal). **Fanghao Lu:** Supervision (equal); validation (equal). **Weihua Zhang:** Funding acquisition (equal); project administration (equal); resources (equal); supervision (equal); validation (equal); writing‐review & editing (equal).

## Supporting information

Supplementary Material

Supplementary Material

## Data Availability

All data generated during this study are included in this published article.
