# Supplementary figures and images for: Hydrogen sulphide ameliorating skeletal muscle atrophy in db/db mice via Muscle RING finger 1 S‐sulfhydration
Source: J Cell Mol Med. 2020 Jul 7;24(16):9362–77. doi: 10.1111/jcmm.15587 (PMC7417732; doi:10.1111/jcmm.15587)

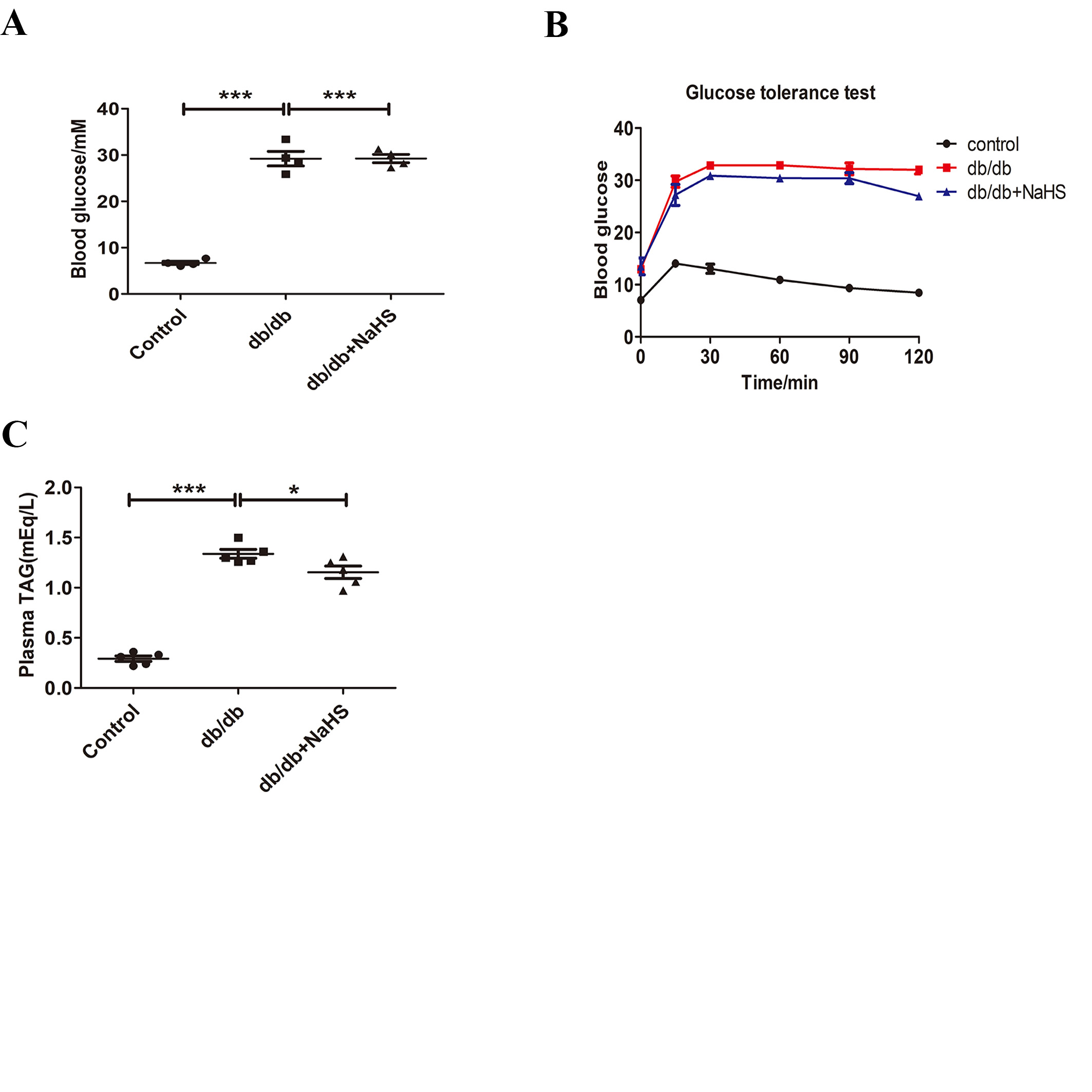

Supplement: Supplementary file 1 — Supplementary Material [file JCMM-24-9362-s001.jpg]

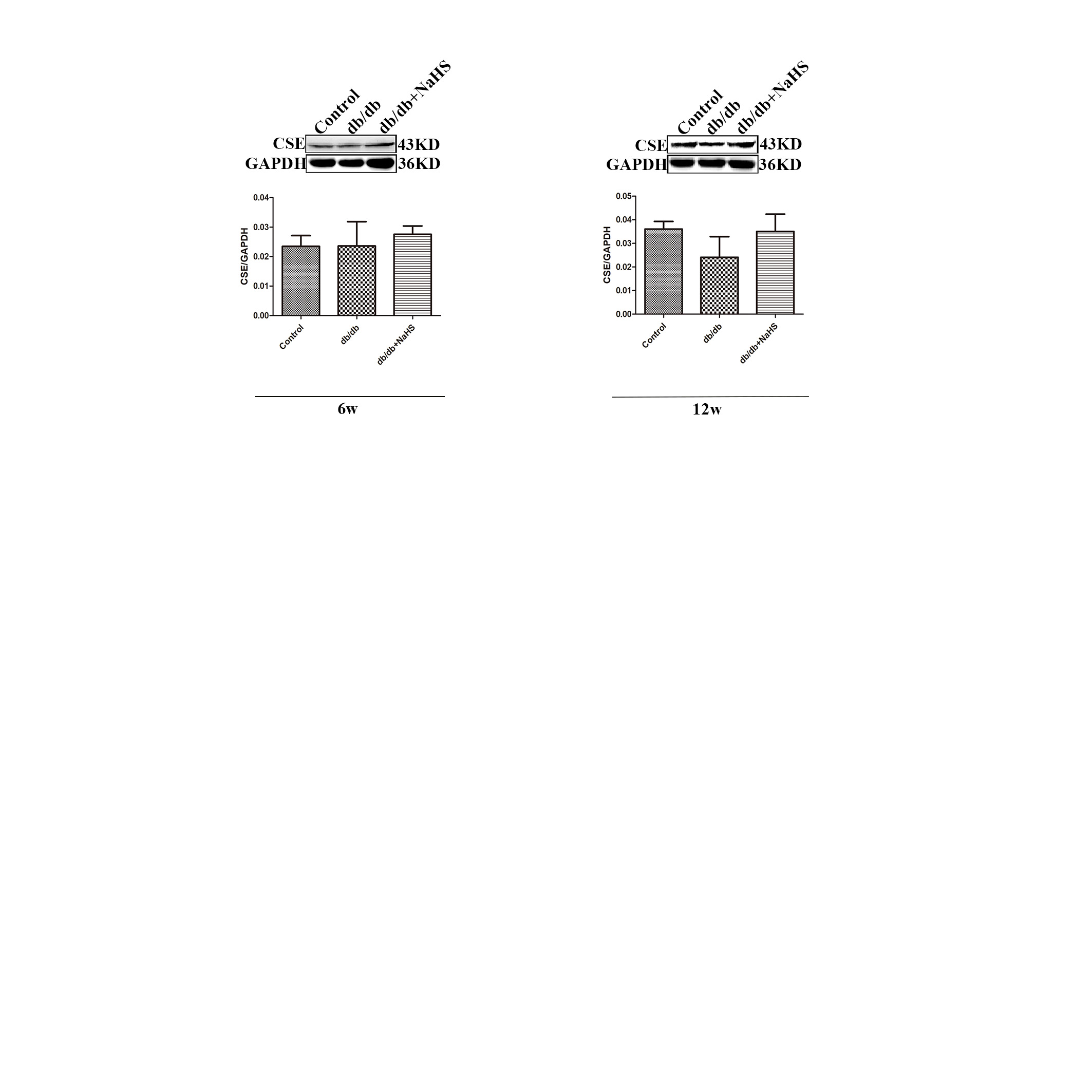

Supplement: Supplementary file 2 — Supplementary Material [file JCMM-24-9362-s002.jpg]
